# The *in vivo* antitumor effects of type I-interferon against hepatocellular carcinoma: the suppression of tumor cell growth and angiogenesis

**DOI:** 10.1038/s41598-017-12414-3

**Published:** 2017-09-22

**Authors:** Hirayuki Enomoto, Lihua Tao, Ryoji Eguchi, Ayuko Sato, Masao Honda, Shuichi Kaneko, Yoshinori Iwata, Hiroki Nishikawa, Hiroyasu Imanishi, Hiroko Iijima, Tohru Tsujimura, Shuhei Nishiguchi

**Affiliations:** 1 Division of Hepatobiliary and Pancreatic Medicine, Department of Internal Medicine, Hyogo College of Medicine, Mukogawa-cho 1-1, Nishinomiya, Hyogo 663-8501 Japan; 2 Department of Pathology, Hyogo College of Medicine, 1-1 Mukogawa-cho, Nishinomiya, Hyogo 663-8501 Japan; 3 Department of Pathology, Medical College of Shantou University, 22 Xinling Road, Shantou City, Guangdong Prov 515041 China; 4 Department of Environmental and Preventive Medicine, Hyogo College of Medicine, 1-1 Mukogawa-cho, Nishinomiya, Hyogo 663-8501 Japan; 5 Department of Gastroenterology, Kanazawa University Hospital, 13-1 Takara-machi, Kanazawa, Ishikawa 920-8641 Japan

## Abstract

Type I-interferon (IFN) is considered to exert antitumor effects through the inhibition of cancer cell proliferation and angiogenesis. Based on the species-specific biological activity of IFN, we evaluated each antitumor mechanism separately. We further examined the antitumor effects of type I-IFN combined with sorafenib. Human IFN (hIFN) significantly inhibited the proliferation of human hepatocellular carcinoma (HCC) Hep3B cells and the tube formation of human umbilical vein endothelial cells (HUVECs) *in vitro*. Although mouse IFN (mIFN) did not inhibit the proliferation of Hep3B cells *in vitro*, mIFN, as well as hIFN, showed significant antitumor effects in mouse Hep3B cell-xenograft model. Furthermore, mIFN treatment amplified the antitumor effects of sorafenib *in vivo* with the suppression of angiogenesis. The DNA chip analysis showed that the mIFN treatment promoted the antitumor signal pathways of sorafenib, including anti-angiogenic effects. Unlike the effects observed in *in vitro* experiments, mIFN showed an antitumor effect in the mouse Hep3B cell-xenograft model, suggesting a role of the anti-angiogenic activity in the *in vivo* tumoricidal effects of type I-IFN. In addition, our findings suggested the clinical utility of combination therapy with type І-IFN and sorafenib for HCC.

## Introduction

Hepatocellular carcinoma (HCC) is the sixth most common tumor and the third cause of cancer-related death. The prognosis of advanced HCC is unfavorable, with a high mortality rate^1,2^.

Sorafenib is an oral multikinase inhibitor that shows antitumor effects by suppressing tumor cell proliferation and angiogenesis through the inhibition of several tyrosine kinases^3,4^. Sorafenib is the first demonstrated agent to improve the median survival and time to progression in patients with advanced HCC^5,6^, although the therapeutic effects of sorafenib on HCC are not obvious in all HCC patients. To achieve better therapeutic efficacy, combination therapies consisting of sorafenib with other drugs have been investigated^7^; however, no combination therapy showing a sufficient clinical effect for HCC treatment has been established.

Interferon (IFN) is known to be a multifunctional molecule exhibiting various biological functions, including antiviral, antiproliferative and immunoregulatory activities, and type І-IFN (IFN-α and IFN-β) has been used for antiviral treatment in patients with chronic hepatitis C in Japan^8,9^. Type I-IFN has also been reported to have antitumor effects in several types of tumors, including HCC^10–14^. Similar to sorafenib, type I-IFN is considered to have tumoricidal effects via two mechanisms: namely, growth inhibitory effects on tumor cells and anti-angiogenetic effects. Recently, experimental studies have reported that combination treatment consisting of sorafenib with human IFN-α (hIFN-α) improves the antitumor effect on HCC both *in vitro* and *in vivo* ^15,16^.

Human tumor xenograft mouse models are generated with the transplantation of human cancer cells into immunodeficient mice; transplanted cancer cells are human cells and cancer stromal cells, including tumor-vascular cells, are derived from mice. Type I-IFN is a highly species-specific molecule, and the cross-reactivity of type I-IFN between human-derived cells and mouse-derived cells is very low^17,18^. The biological activity of mouse IFN (mIFN) on human cells is estimated to be less than 10^−4^ the level of that of hIFN. In addition, the biological activity of hIFN on mouse cells is estimated to be less than 10^−4^ the level of that of mIFN. Since hIFN barely acts on mouse cells, the antitumor effects of hIFN in mouse xenograft models mainly depend on the growth inhibitory effects on human cancer cells. Therefore, it is difficult to adequately assess anti-angiogenic effects *in vivo* mouse xenograft models using hIFN treatment. In contrast, the administration of mIFN acts on mouse cancer stromal cells, suggesting that its antitumor effects are mainly caused by the inhibition of angiogenesis. Therefore, the highly specific (species-dependent) activity of IFN allows us to investigate two different antitumor mechanisms (anti-proliferation and anti-angiogenesis) separately.

In the present study, we first investigated the functional role of type I-IFN in the proliferation of human HCC Hep3B cells and angiogenesis of human umbilical vein endothelial cells (HUVECs). We next administered hIFN and mIFN to mice in a xenograft model and compared the antitumor effects *in vivo*. In addition, we examined the antitumor effects of the combination of sorafenib and type I-IFN.

## Results

### Inhibition of Hep3B cell proliferation and HUVEC tube formation by type І-IFN *in vitro*

**Figure 1:**
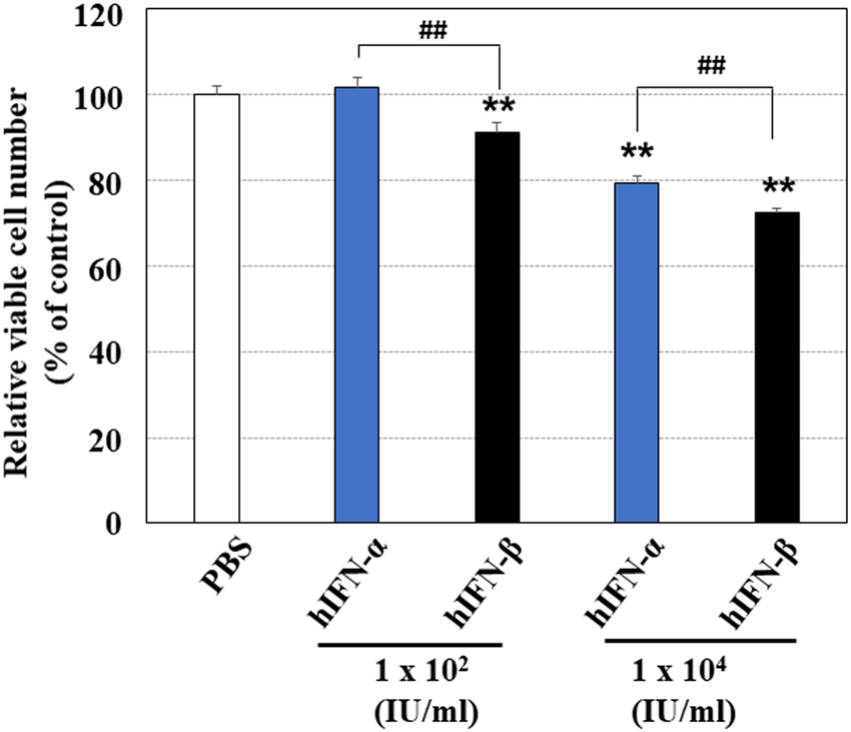
Effects of hIFN-α and hIFN-β on the proliferation of human hepatocellular carcinoma Hep3B cells. Human HCC Hep3B cells were treated with either hIFN-α or hIFN-β for 72 hours. Both hIFN-α and hIFN-β suppressed the proliferation of Hep3B cells; however, the anti-proliferative effect of hIFN-β was more evident than that of hIFN-α. **P < 0.01 versus control (PBS treatment). hIFN-β treatment resulted in a significantly lower cell number than the hIFN-α treatment (^##^P < 0.01).

**Figure 2:**
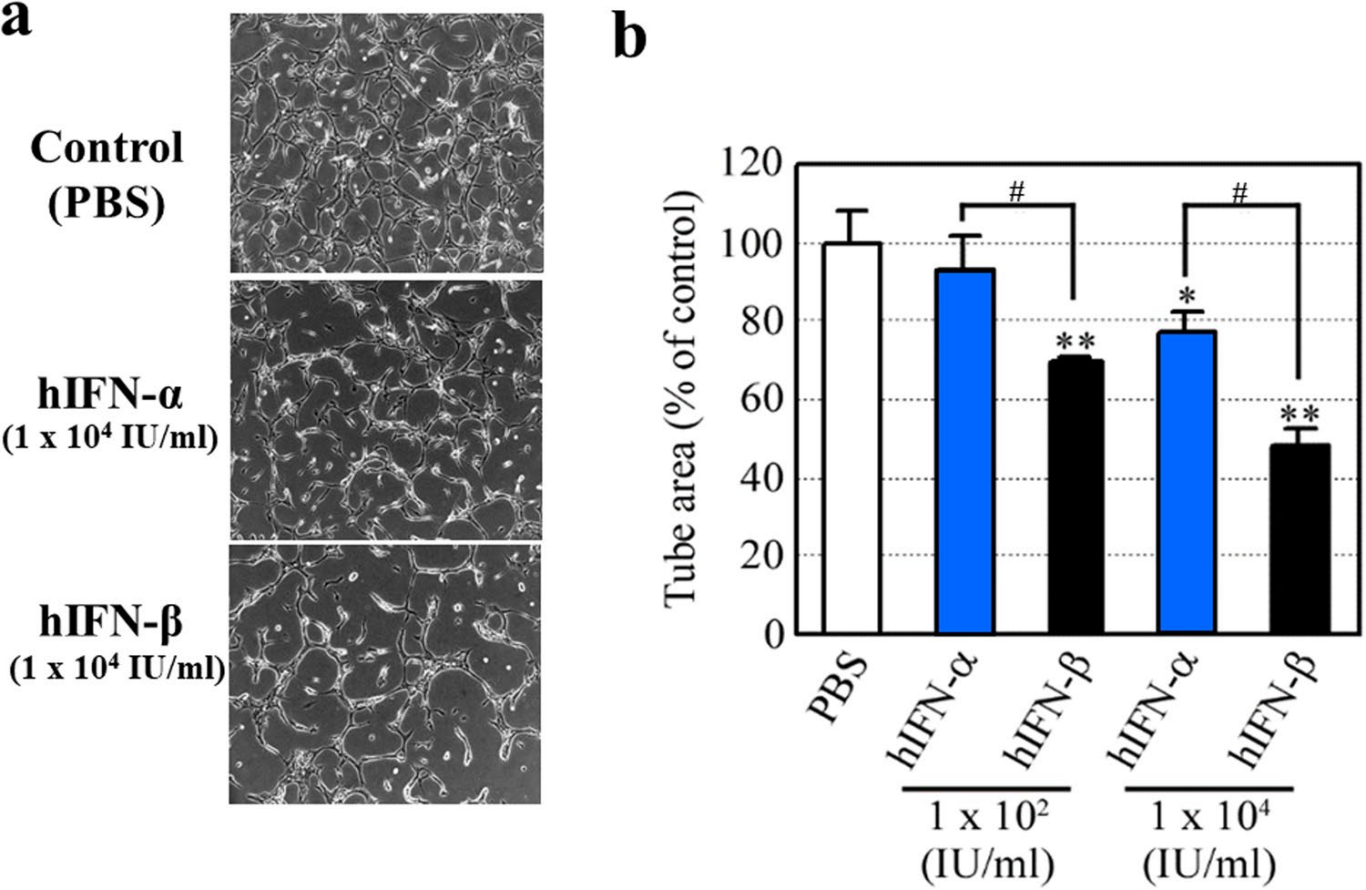
Effects of hIFN-α and hIFN-β on the tube formation of human umbilical vein endothelial cells (HUVECs). HUVECs were treated with either hIFN-α or hIFN-β for 24 hours and a tube formation assay was performed. (**a**) Representative photographs are shown. (**b**) Both hIFN-α and hIFN-β suppressed the tube formation of HUVECs *in vitro*; however, the effects of hIFN-β were more evident than that of IFN-α. *P < 0.05 and **P < 0.01 versus control (PBS treatment). hIFN-β treatment resulted in a significantly smaller area of tube formation compared with the hIFN-α treatment (^#^P < 0.05).

The inhibition of cancer cell proliferation and angiogenesis is considered to be a major mechanism for the tumoricidal effect of type I-IFN (IFN-α and IFN-β). Since it has been reported that the antitumor effects of hIFN-β are higher than those of hIFN-α^19,20^, we first compared the tumoricidal effects of hIFN-α with those of hIFN-β *in vitro*. Both hIFN-α and hIFN-β suppressed the proliferation of human Hep3B cells; however, the effects were more evident when the cells were treated with hIFN-β than with hIFN-α (Fig. 1). In addition, although both hIFN-α and hIFN-β also inhibited the tube formation of HUVECs *in vitro*, hIFN-β showed higher inhibitory effects than hIFN-α (Fig. 2). These findings suggest that hIFN-β inhibits tumor cell proliferation and angiogenesis more effectively in HCC than hIFN-α.

### Antitumor effects of hIFN-β and mIFN-β *in vitro* and *in vivo*

**Figure 3:**
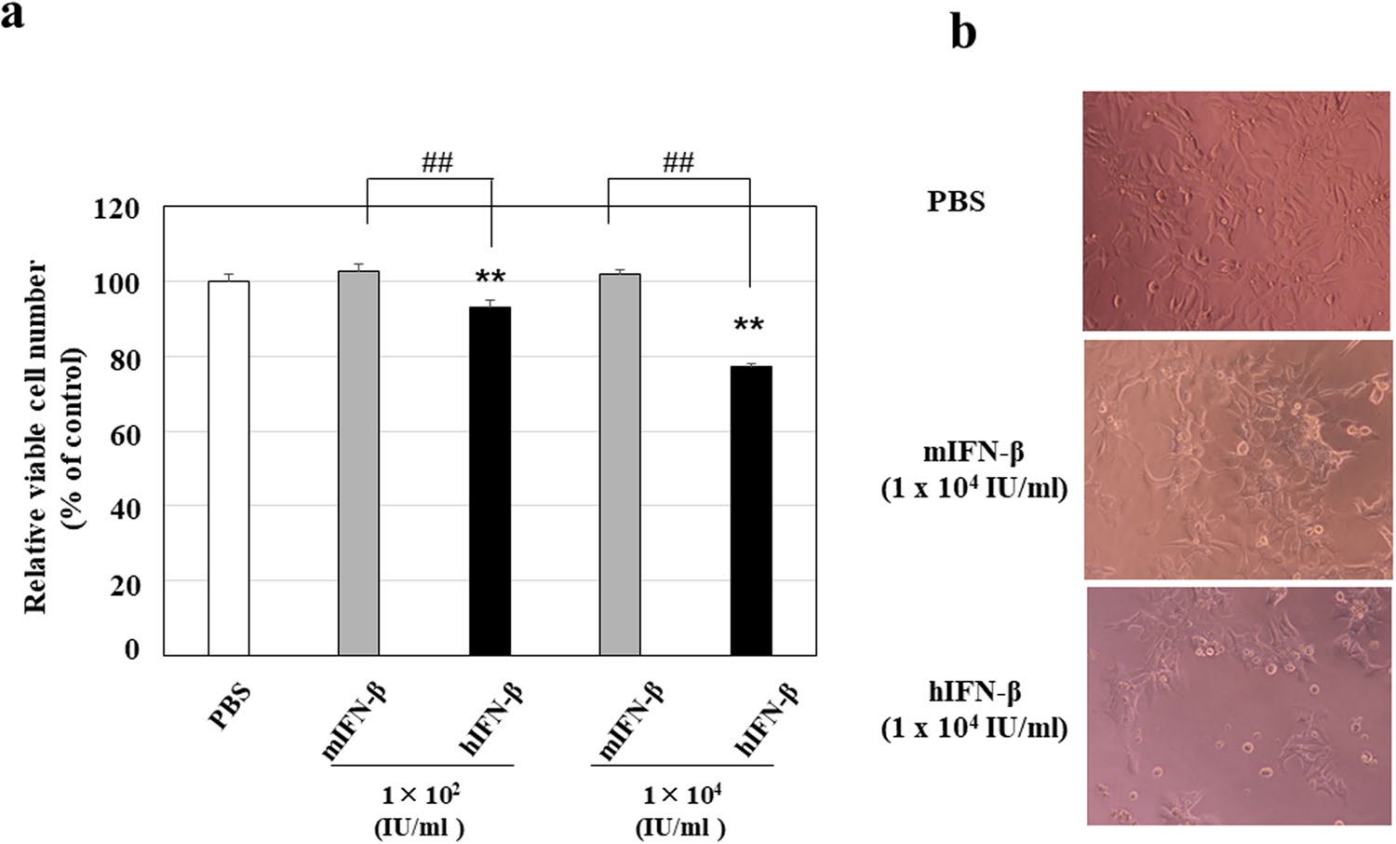
Effects of hIFN-β and mIFN-β on the proliferation of Hep3B cells *in vitro*. (**a**) Hep3B cells were cultured in the presence of hIFN-β or mIFN-β for 72 hours and the viable cell number was measured. The anti-proliferative effect of hIFN-β was more evident than that of mIFN-β (^##^P < 0.01). **P < 0.01 versus control (PBS treatment). (**b**) Representative photographs are shown.

**Figure 4:**
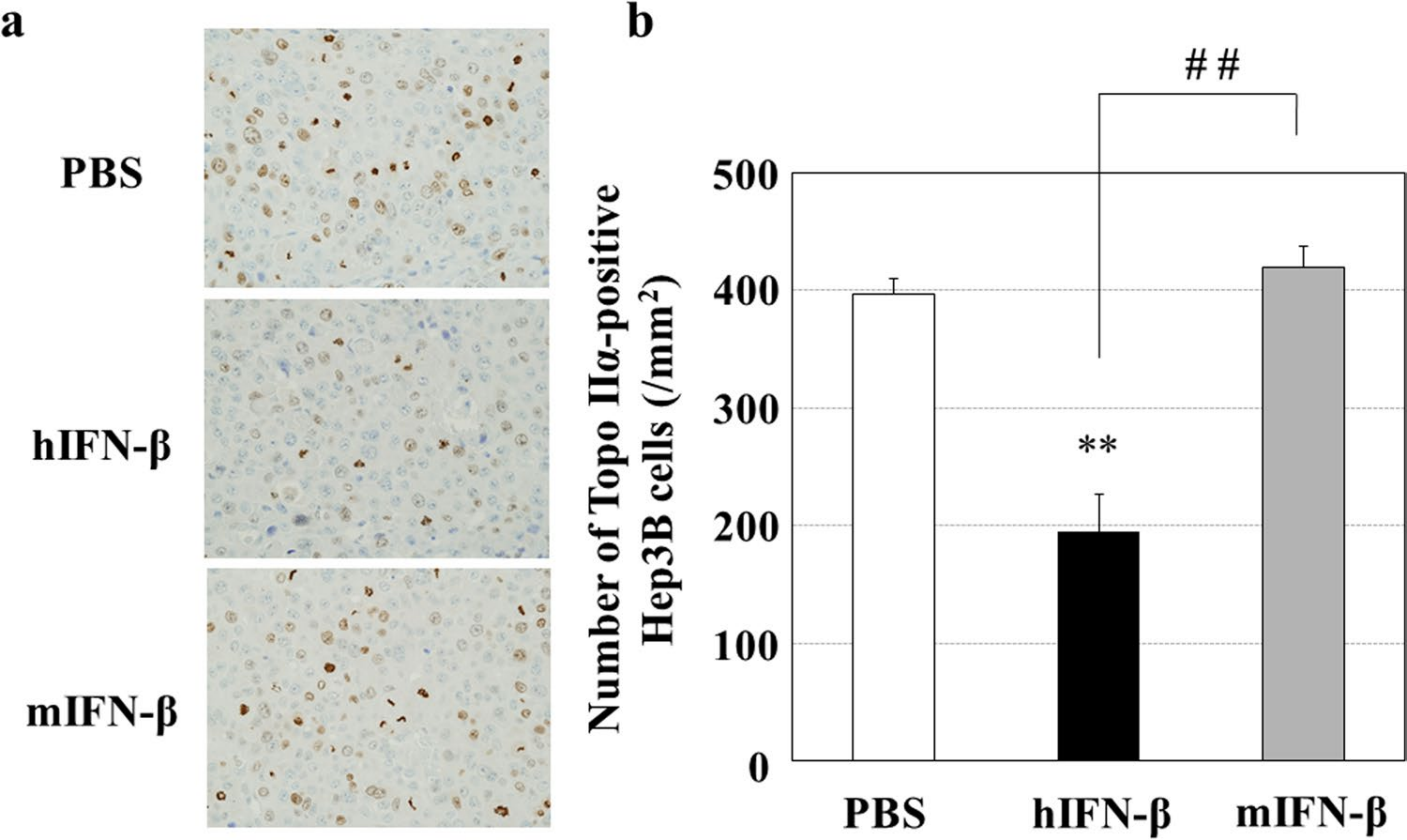
Effects of hIFN-β and mIFN-β on the mitosis of human Hep3B cells *in vivo*. Five weeks after inoculation of Hep3B cells, tumor-bearing mice were randomized into three groups (PBS control, hIFN-β treatment and mIFN-β treatment), and daily peritumoral injections of PBS, hIFN-β or mIFN-β (1 × 10^4^ IU/site) were performed for two weeks. The Hep3B tumors that developed in the mouse xenograft model were used for the histological evaluation (N = 6 per each group). (**a**) Sections of Hep3B tumors were immunostained with an anti-human topoisomerase IIα (Topo IIα) antibody, and representative photographs are shown. (**b**) The number of Topo-IIα-positive Hep3B cells were counted in 10 areas selected randomly in each specimen of tumors. In agreement with the *in vitro* study, the number of proliferating HepB cells decreased with the treatment of hIFN-β significantly *in vivo*, while mIFN-β did not affect the number of proliferating Hep3B cells. ** P < 0.01 versus control (PBS treatment) and ^##^P < 0.01 between the groups.

**Figure 5:**
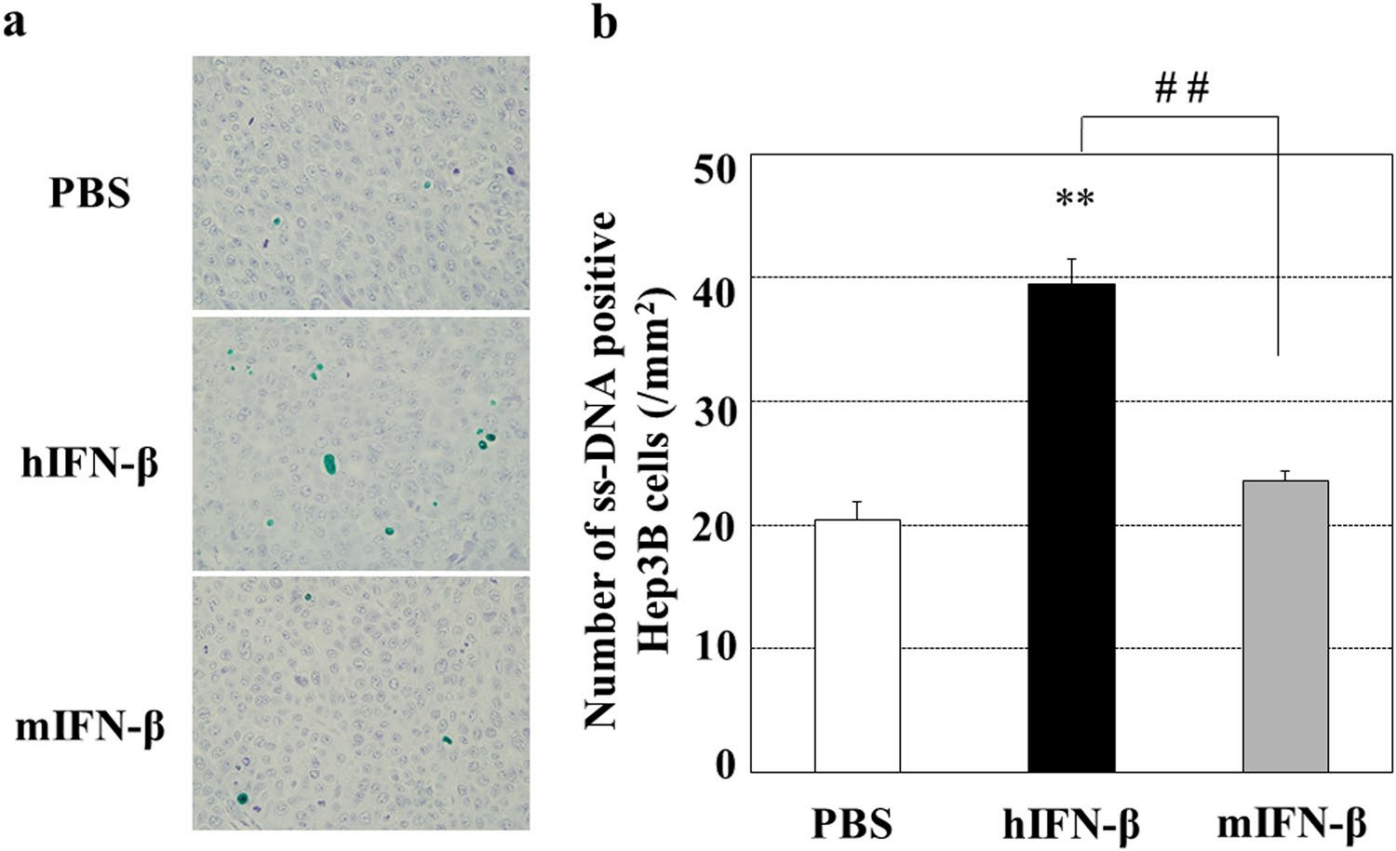
Apoptosis induction of hIFN-β and mIFN-β in human Hep3B cells *in vivo*. The Hep3B tumors shown in Fig. 4 were used for the detection of single stranded DNA (ssDNA)-positive Hep3B cells. (**a**) Sections of Hep3B tumors were immunostained with an anti-ssDNA antibody, and apoptotic cells were detected with green signals of HistoGreen. Representative photographs are shown. (**b**) The number of ssDNA-positive Hep3B cells were counted in 10 areas selected randomly in each specimen of tumors. Treatment with hIFN–β significantly induced apoptosis in the Hep3B cells *in vivo*, whereas that with mIFN-β did not. **P < 0.01 versus control (PBS treatment) and ^##^P < 0.01 between the groups.

**Figure 6:**
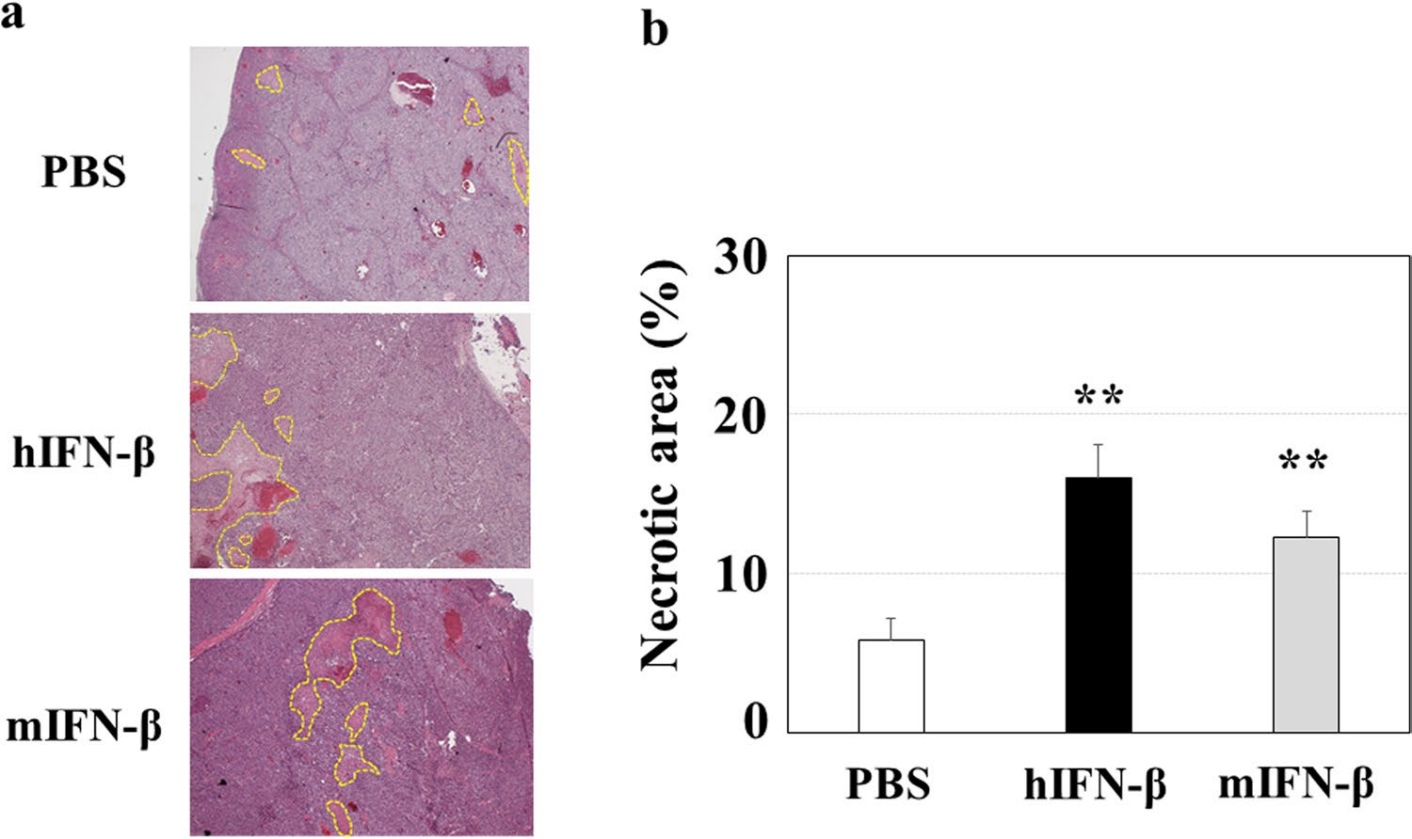
Effects of hIFN-β and mIFN-β in the *in vivo* mouse xenograft model. The Hep3B tumors shown in Figs 4 and 5 were used for the histological evaluation. (**a**) Sections of Hep3B tumors were stained with hematoxylin-eosin, and representative photographs are shown. Necrotic tissues are shown as the areas surrounded with the dot lines. (**b**) Treatment with mIFN-β caused increased areas of necrotic tissue versus that seen in the tumors in the control (PBS treatment) mice. **P < 0.01 versus control (PBS treatment).

**Figure 7:**
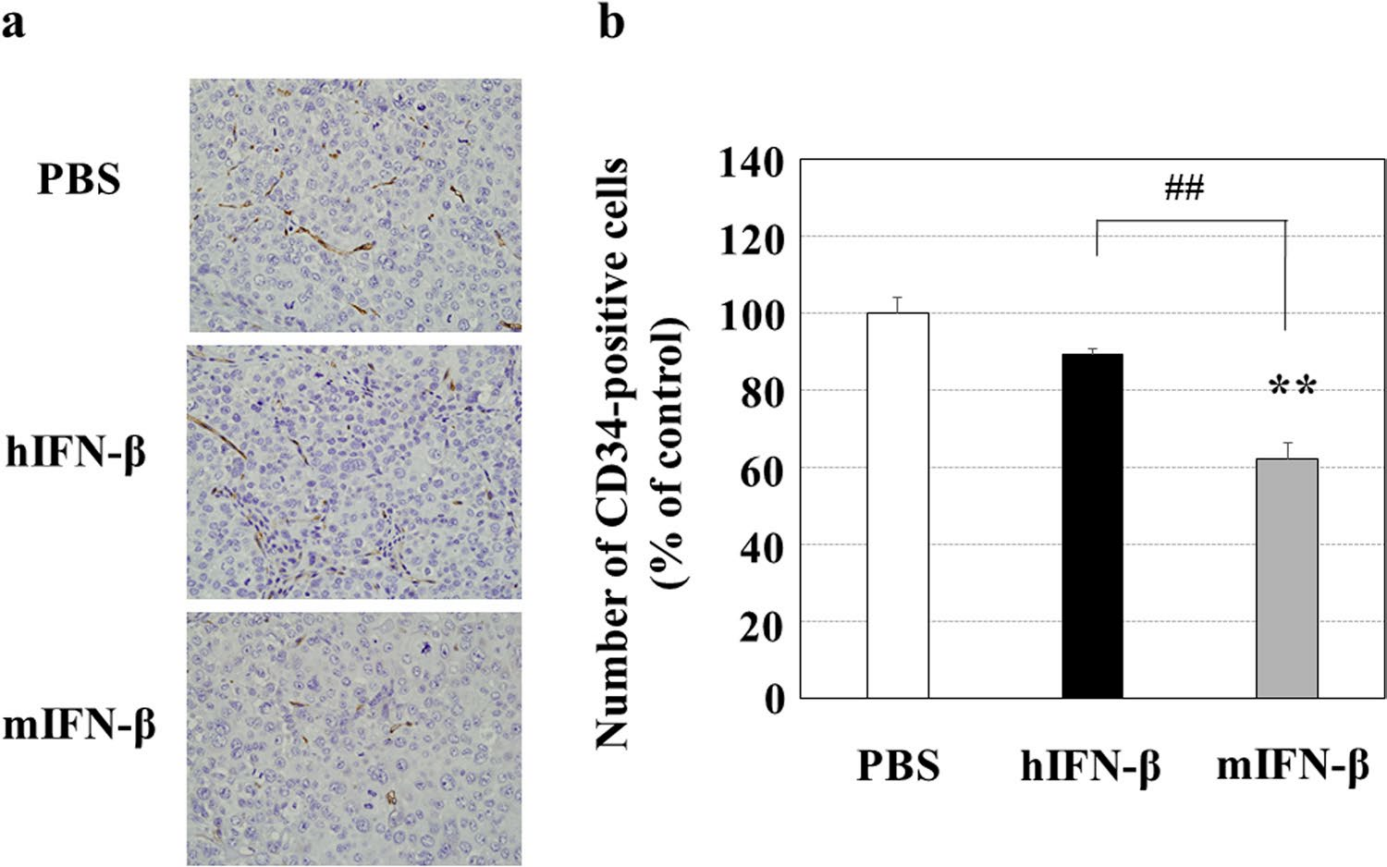
Anti-angiogenic effects of hIFN-β and mIFN-β in the *in vivo* mouse xenograft model. The Hep3B tumors shown in Figs 4 and 5 were also used for the evaluation of anti-angiogenic effects. (**a**) Sections of Hep3B tumors were immunostained with an anti-mouse CD34 antibody, and CD34-positive cells were detected with brown signals of DAB. Representative photographs are shown. (**b**) The number of CD34-positive cells were counted in 10 areas selected randomly in each specimen of tumors. The number of CD34-positive cells was significantly lower in the tumors treated with mIFN-β than in the tumors treated with PBS and hIFN-β. **P < 0.01 versus control (PBS treatment) and ^##^P < 0.01 between the groups.

Since hIFN-β inhibited the proliferation of human Hep3B cells and the tube formation of HUVECs more successfully than hIFN-α *in vitro*, we selected IFN-β to investigate the antitumor effects of type І-IFN. Because of the species-specific biological activity of type I-IFN, the *in vitro* proliferation inhibitory effect of hIFN-β on human Hep3B cells was higher than that of mIFN-β (Fig. 3). In agreement with the *in vitro* study, when we performed *in vivo* study with the mouse Hep3B cell-xenograft model, hIFN-β decreased the number of topoisomerase IIα (Topo IIα)-positive human Hep3B cells, whereas mIFN-β did not (Fig. 4). Since Topo IIα functions during late G2 and M-phase of the cell cycle, these results indicate that hIFN-β suppresses the proliferation (mitotic activity) of Hep3B cells. In addition, hIFN-β increased the number of single stranded DNA (ssDNA)-positive human Hep3B cells, while mIFN-β did not, indicating that hIFN-β induces the apoptosis in Hep3B cells (Fig. 5). However, in the *in vivo* study with the mouse Hep3B cell-xenograft model, mIFN-β treatment showed significant tumoricidal effects, which were almost equivalent to those of hIFN-β treatment (Fig. 6). In the mouse Hep3B cell-xenograft model, an immunohistochemical study of CD34 revealed that the number of CD34-positive cells in Hep3B tumors treated with mIFN-β was decreased when compared to that seen in the Hep3B tumors treated with hIFN-β, indicating that tumor vascular formation was more severely suppressed with mIFN-β treatment than with hIFN-β treatment (Fig. 7). These findings suggest that the action on cancer stromal cells has an important role in the *in vivo* tumoricidal effect of type I-IFN as well as the direct inhibition of cancer cell proliferation.

### Antitumor effects of combination therapy with sorafenib plus mIFN-β *in vivo*

**Figure 8:**
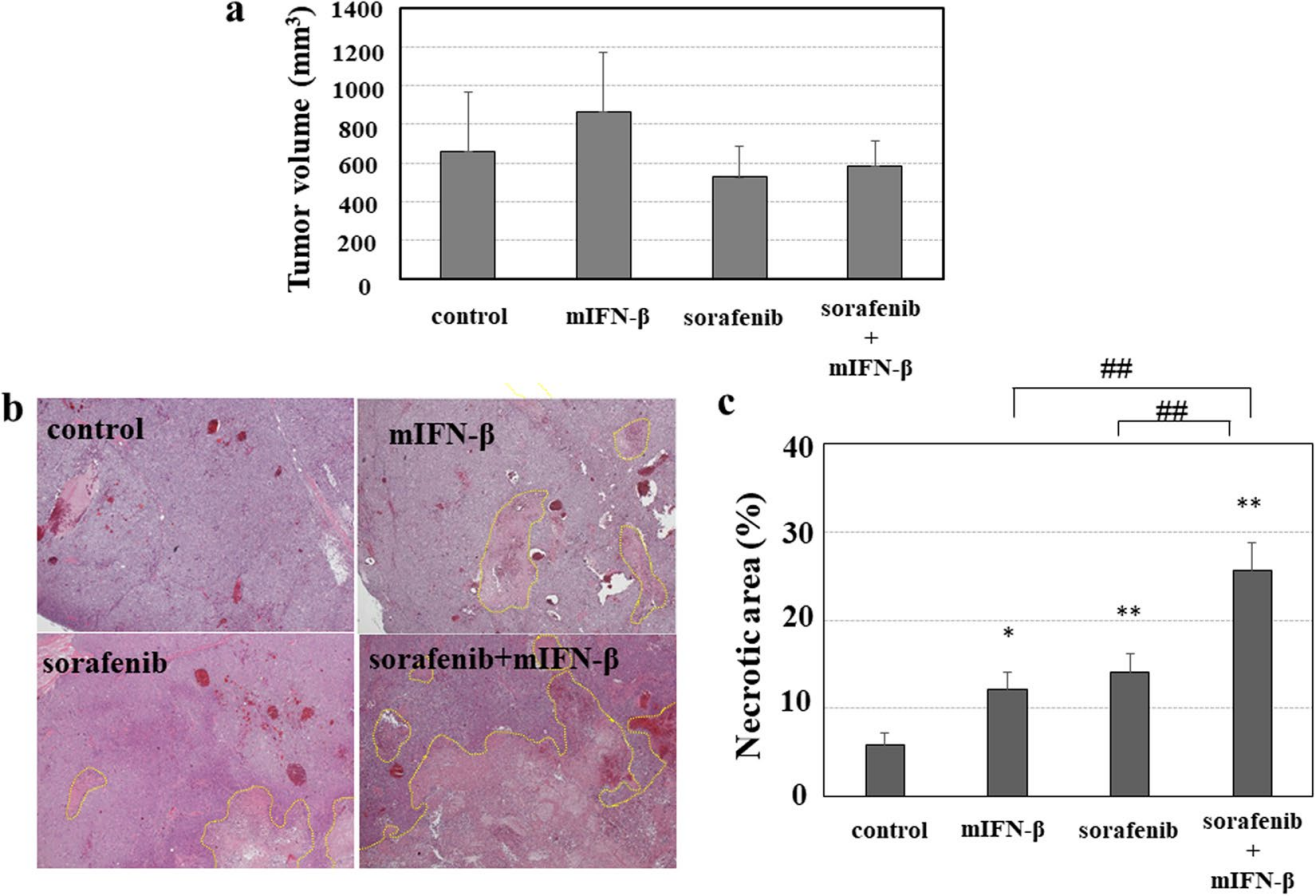
*In vivo* antitumor effects of sorafenib plus mIFN-β treatment in the mouse xenograft model. Five weeks after inoculation of Hep3B cells, tumor-bearing mice were randomized into four groups (PBS control, mIFN-β treatment, sorafenib treatment and sorafenib plus mIFN-β treatment). Sorafenib (30 mg/kg) was administered orally for two weeks. PBS or mIFN-β (1 × 10^4^ IU/site) was administered via daily peritumoral injection for two weeks. The Hep3B tumors that developed in the mouse xenograft model were used for the histological evaluation (N = 6 per each group). (**a**) The tumor volume in the mouse Hep3B cell-xenograft model was not significantly different among the four groups. (**b**) Sections of Hep3B tumors were stained with hematoxylin-eosin. The necrotic areas are surrounded by the dot lines. (**c**) The necrotic areas were measured in 10 sections randomly selected in each specimen of tumors. Treatment with mIFN-β or sorafenib resulted in increased the necrotic areas compared with that observed in the tumors that developed in the control (PBS treatment) mice (*P < 0.05 and **P < 0.01). Combination therapy with sorafenib plus mIFN-β showed more evident tumor necrotic effects than treatment with mIFN-β or sorafenib alone (^##^P < 0.01).

**Figure 9:**
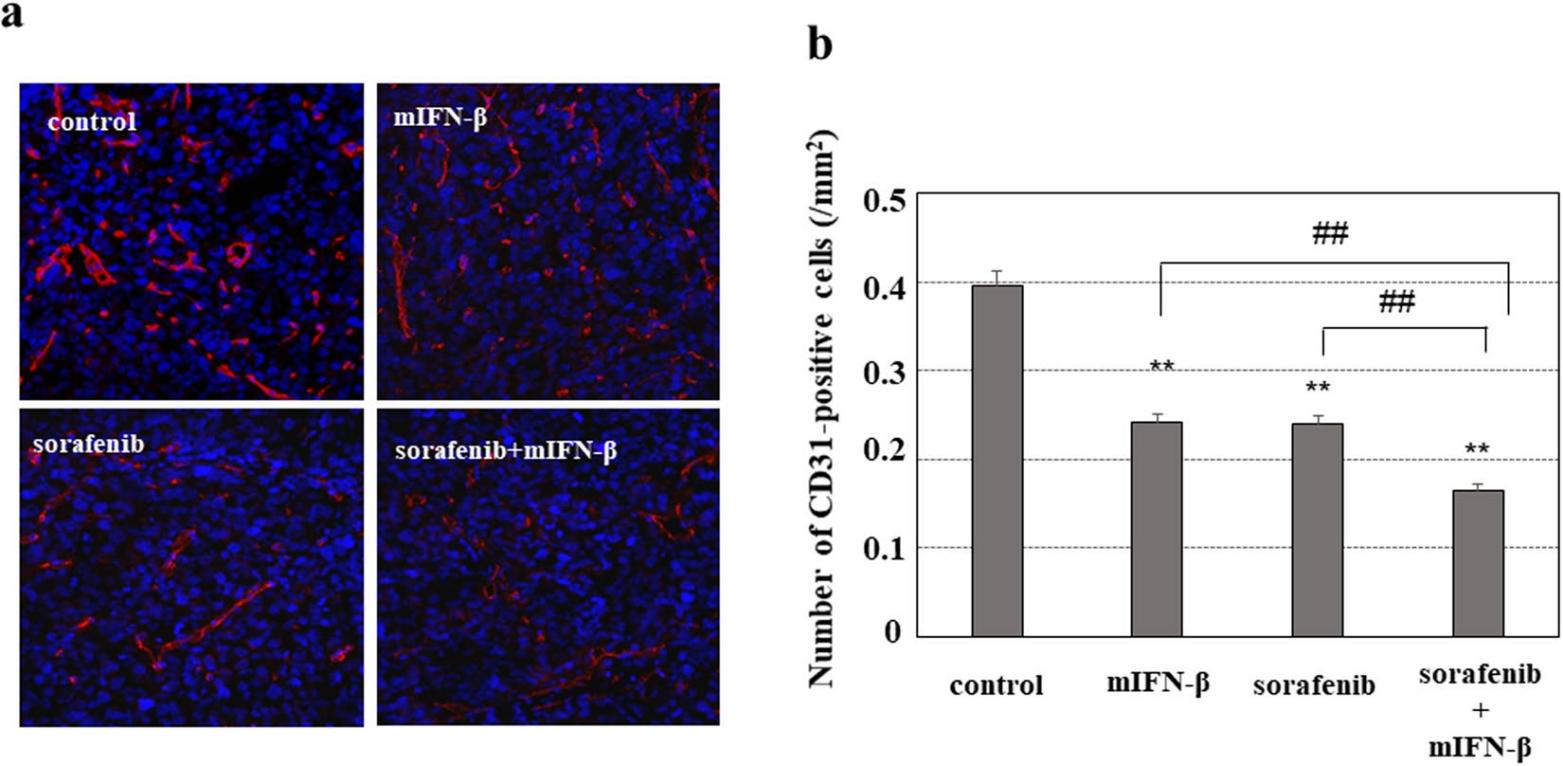
*In vivo* anti-angiogenic effects of sorafenib plus mIFN-β treatment in the mouse xenograft model. The Hep3B tumors shown in Fig. 8 were also used for the evaluation of anti-angiogenic effects. (**a**) Sections of Hep3B tumors were immunostained with an anti-mouse CD31 antibody and positive signals were visualized with fluorescence using Alexa Fluor546. Representative photographs are shown. (**b**) CD31-positive cells were counted in more than 8 areas selected randomly in each specimen of tumors. The tumors treated with mIFN-β or sorafenib showed a decreased number of CD31-positive cells compared with the tumors that developed in the control (PBS treatment) mice (**P < 0.01). In addition, combination therapy with sorafenib plus mIFN-β resulted in a decreased number of CD31-positive cells versus treatment with mIFN-β or sorafenib alone (^##^P < 0.01).

Sorafenib is widely used as an established medicine that targets tumor angiogenesis and shows a significant antitumor effect in clinical practice. Based on the suggested significant antitumor role of the anti-angiogenic effects of type I-IFN as shown above, we next investigated the efficacy of combination therapy with sorafenib and type I-IFN as a new anti-angiogenic therapy for HCC *in vivo*. Although the tumor size was not significantly different among the four groups (PBS control, mIFN-β alone, sorafenib alone and sorafenib plus mIFN-β) (Fig. 8a), both mIFN-β and sorafenib monotherapy increased the necrotic areas of Hep3B tumors in the mouse xenograft model. In addition, necrotic areas were further enlarged with the combination of sorafenib plus mIFN-β (Fig. 8b and c). When we evaluated the effects of sorafenib and mIFN-β on the development of tumor blood vessels, the anti-angiogenic effects were accelerated by sorafenib plus mIFN-β therapy compared with sorafenib or mIFN-β monotherapy (Fig. 9). These findings suggest that type I-IFN exaggerates the antitumor effects of sorafenib by increasing its anti-angiogenic effects.

In order to assess how mIFN-β accelerates the *in vivo* antitumor effects of sorafenib, we compared the gene expression profiles in developed Hep3B tumors (which included tumor cells and stromal cells) receiving sorafenib plus mIFN-β treatment and compared with those noted in Hep3B tumors receiving sorafenib treatment alone in the mouse xenograft model. Although the expression changes for each gene were small, perhaps because various types of cells were included in a given tumor, a pathway analysis revealed significant changes in 22 pathways of the 167 pathways examined. Among the 22 pathways, 13 signal pathways were significantly decreased and nine pathways were significantly increased (Table 1). Pathways with decreased gene expressions included those related to angiogenesis or cellular proliferation, such as VEGF signaling pathway, mitogen-activated protein kinase (MAPK) signaling pathway and insulin signaling pathway, whereas expressions of genes related to apoptosis were significantly increased. These results showed that mIFN, which is thought to act on mouse cancer stromal cells, increases the antitumor effects of sorafenib through several mechanisms, including amplified anti-angiogenic effects.

**Table 1:** Pathway analysis: Comparison of gene expression between tumors treated with sorafenib plus type I-IFN treatment and those treated with sorafenib alone.

Name of pathway	Number of genes	P-value (GSA test)
**Up-regulated pathway in sorafenib plus type I-IFN treatment**		
Apoptosis	81	<0.005
Toll-like receptor signaling pathway	65	<0.005
Tyrosine metabolism	58	<0.005
Metabolism of xenobiotics by cytochrome P450	57	<0.005
Aminosugars metabolism	31	<0.005
Alkaloid biosynthesis II	21	<0.005
Androgen and estrogen metabolism	46	0.04
Glutathione metabolism	43	0.04
RNA polymerase	26	0.04
**Down-regulated pathways in sorafenib plus type I-IFN treatment**		
Cytokine-cytokine receptor interaction	175	<0.005
Insulin signaling pathway	136	<0.005
Colorectal cancer	84	<0.005
Adherens junction	80	<0.005
Fc epsilon RI signaling pathway	70	<0.005
Inositol phosphate metabolism	46	<0.005
Type II diabetes mellitus	43	<0.005
Streptomycin biosynthesis	10	<0.005
2,4-Dichlorobenzoate degradation	5	<0.005
MAPK signaling pathway	270	0.035
VEGF signaling pathway	71	0.035
T cell receptor signaling pathway	81	0.04
Styrene degradation	5	0.045

The result is included in the microarray data (GSE102863).

## Discussion

Sorafenib is an oral multikinase inhibitor with an antitumor activity against various types of cancers^6^. We investigated the possibility of using the combination of sorafenib and type I-IFN as a new anti-angiogenic therapy for HCC. One unique point in this study is that we investigated the antitumor effect of mIFN-β compared with that of hIFN-β *in vivo*. Although mIFN-β did not directly inhibit the proliferation of human Hep3B cells *in vitro* (Fig. 3), mIFN-β, as well as hIFN-β, showed significant therapeutic efficacy in the mouse Hep3B cell-xenograft model, suggesting the important role of the anti-angiogenic activity in the tumoricidal effects of type I-IFN *in vivo*.

Type І-IFN (hIFN-α and hIFN-β) is clinically used for antiviral treatment in Japan. In the present study, we investigated the biological effects of two type І-IFN subsets (hIFN-α and hIFN-β) on human HCC Hep3B cells and HUVEC cells. Since hIFN-β showed higher inhibitory effects on both Hep3B cell proliferation (Fig. 1) and HUVEC tube formation (Fig. 2) than those of hIFN-α *in vitro*, we used IFN-β in the *in vivo* studies. Among type I-IFN subsets, IFN-α and IFN-β have been reported to exert different anti-angiogenic activities *in vitro* ^19,21,22^, and Noguchi *et al*.^19^ showed a more potent inhibitory effect of hIFN-β on endothelial cell proliferation than hIFN-α *in vitro*. Our findings are consistent with these reports and also indicate the high antitumor effects of IFN-β.

Combination therapy with sorafenib plus mIFN-β increased the necrotic areas in the Hep3B tumors in the mouse xenograft model compared with treatment with sorafenib or IFN-β alone. In the present study, we used Hep3B cells to evaluate antitumor effects of type-IFN on HCC cells. In addition, we also found the antitumor effects of hIFN-β by use of a different HCC cell line, HepG2 *in vitro* (Supplementary Fig. S1). Combination therapy with sorafenib plus mIFN-β resulted in significant increase in necrotic area (Supplementary Fig. S2) and decrease in number of CD31-positive cells in HepG2 tumors (Supplementary Fig. S3). These results support the findings of mouse Hep3B cell-xenograft model. Furthermore, antitumor effects of type I-IFN on various HCC cell lines, including Huh-7 cells^15^, HAK-1B cells^16^ and KIM-1 cells^16^, have been also reported, suggesting the significant antitumor effects of type I-IFN on HCC cells *in vitro* and *in vivo*.

In the present study, no significant differences were observed among the four groups (PBS control, mIFN-β alone, sorafenib alone, and sorafenib plus mIFN-β) in the Hep3B tumor volume (Fig. 8a) and HepG2 tumor volume (Supplementary Fig. S2a). However, unlike our results, previous studies have shown that sorafenib and/or hIFN-α treatment reduces the volume of tumors formed in mice when compared with their control counterparts^15,16^. Although we did not clarify the reasons for the different findings between previous and the current study, the discrepancies may depend on the different cell lines or recipient mice used in the studies. Additionally, the difference in type I-IFN selected in each study may have influenced the results. Previous studies suggested that the effects of sorafenib on the cellular proliferation and survival *in vivo* tumors were different depending on the kind of transplanted cells^15,16^. With regard to our experimental conditions, sorafenib monotherapy showed only mild effects on hepatoma cells (Supplementary Table S1). Therefore, under the current *in vivo* experimental conditions, the combination therapy (sorafenib plus mIFN) should mainly act on cancer stromal cells rather than hepatoma cells. These findings may explain the reason why the combination therapy with sorafenib plus mIFN-β resulted in significant increase in the necrotic area and decrease in the number of CD31-positive cells without a significant reduction of the tumor volume.

The DNA-Chip analysis showed that mIFN-β increased the antitumor effects of sorafenib via several mechanisms, including the decreased angiogenetic signal and the increased apoptotic signal (Table 1). In the present study, we assessed *in vivo* anti-angiogenetic effects by counting the number of CD34- and CD31-positive endothelial cells (Figs 7 and 9). Although it is not easy to evaluate the functional changes of endothelial cells in mouse tumors, we found significant antitumor effects of mIFN-β without actions on human Hep3B cells (Figs 4 and 5), thus suggesting that type I-IFN is able to affect functions of cancer stromal cells, including endothelial cells *in vivo*. We herein showed that type-I IFNs inhibited the tube formation of HUVECs (Fig. 2) *in vitro*. Additionally, we have found that type-І hIFN is able to induce the apoptosis of HUVECs *in vitro* (Supplementary Fig. S4). These findings suggest that type-І IFNs may affect the functions of endothelial cells. Anti-angiogenic effects caused by the decreased the number and the functional change of endothelial cells by the mIFN-β treatment may be involved in the enlargement of necrotic areas induced *in vivo*, although we were unable to completely clarify the detailed antitumor mechanisms regarding combination therapy with sorafenib and type-І IFNs for HCC. Our data suggested that anti-angiogenic effects should participate in antitumor effects *in vivo*, but we cannot deny some additional mechanism(s) may be also involved.

Our experimental studies regarding the comparison of two type I-IFN subsets (hIFN-β and mIFN-β) revealed that the anti-angiogenic activity may have an important role in the antitumor effects of type I-IFN *in vivo*. Since type-I hIFN acts on both cancer cells and cancer stromal cells in clinical practice, this combination therapy may provide a more evident antitumor effect for HCC treatment. Indeed, a preliminary clinical study by Itokawa *et al*.^23^ showed the possible clinical utility of the combination therapy with sorafenib and interferon in advanced HCC patients.

Recently, the application of sorafenib with other medical drugs has been investigated, and combination therapy consisting of sorafenib with hIFN-α is available for patients with metastatic renal cell carcinoma (RCC)^24,25^. Tochizawa *et al*.^26^ investigated the antitumor effects of sorafenib plus IFN-α on various RCC cell lines and showed that the growth of six RCC cell lines were more effectively suppressed with the treatment of the sorafenib plus IFN-α than the those of IFN-α or sorafenib alone. However, the intracellular signal transduction such as the activation of MAPK cascades were different in each cell line. Although results of experimental studies, including ours, suggest the efficacy of combination therapy for HCC as well as RCC, additional basic studies are necessary for the clarification of the detailed mechanisms.

In summary, we found that mIFN-β, which specifically acts on cancer stromal cells, showed significant antitumor effects on human HCC Hep3B cell-xenograft model, thus suggesting the important role of anti-angiogenic effects in the tumoricidal effects of type І-IFN *in vivo*. Our findings also suggest the clinical utility of combination therapy with type І-IFN and sorafenib for HCC, similar to previously established effective therapies for RCC.

## Materials and Methods

### Reagents

The human HCC cell line, Hep3B, and HUVECs were obtained from the American Type Culture Collection (Manassas, VA) and Lonza (Walkersville, MD), respectively. NOD.CB-17Prkdc scid/J mice were purchased from Charles River Laboratories Japan (Yokohama, Japan). IFN-α and IFN-β were kindly provided by Dainippon Sumitomo Pharma Co., Ltd (Osaka, Japan) and Toray Industries (Tokyo, Japan), respectively. Sorafenib was purchased from Bayer Pharmaceutical Corporation. Sorafenib was dissolved in dimethyl sulfoxide to create a 10 mM stock solution. The dissolved solutions were stored at −20 °C for the *in vitro* studies. With regard to the *in vivo* studies, we prepared the solution at the time of usage.

### Cell culture and collagen-gel sandwich tube-formation assay

Hep3B cells were cultured in Dulbecco’s modified Eagle’s medium (DMEM) containing 10% fetal bovine serum (FBS) in 5% CO_2_ at 37 °C and treated with 1 × 10^2^–1 × 10^4^ IU/ml of hIFN-α or hIFN-β. The cells were cultured in the presence of hIFN-α or hIFN-β at various concentrations. HUVECs were cultured between two collagen gel layers in the presence of the indicated concentrations of hIFN, and the degree of capillary-like tube formation was quantified by determining the pixel number of tubes on each image, as reported previously^27^.

### DNA synthesis and cell proliferation assay

For the cell proliferation assay, cells were plated onto 96-well plates at a density of 3 × 10^3^ cells/well in DMEM supplemented with 10% FBS. After 24 hours, the cells were given fresh medium supplemented with 10% FBS. The cells were further cultured in the medium for an additional 72 hours in the presence or absence of the indicated type І-IFN (hIFN-α, hIFN-β or mIFN-β), and the cell proliferation was estimated using the colorimetric assay method with the Cell Counting Kit (Dojindo, Kumamoto, Japan) for the indicated time^28,29^.

### Mouse Hep3B cell-xenograft model and histological assessments

A mouse Hep3B cell-xenograft model was generated according to previously described methods^30,31^. In brief, Hep3B cells were inoculated into both flanks (1 × 10^7^cells/flank). One day before the initiation of IFN-β treatment, pretreatment with anti-asialo-GM1 polyclonal antisera 25% (v/v) i.p. was performed for NK cell depletion. After five weeks, in order to compare the antitumor effects between hIFN-β and mIFN-β, tumor-bearing mice (~8 mm in size) were randomized into three groups (PBS control, hIFN-β treatment and mIFN-β treatment), and daily peritumoral injections of hIFN-β or mIFN-β (1 × 10^4^ IU/site) were performed for two weeks.

To investigate the antitumor effects of sorafenib and mIFN-β, the tumor-bearing mice were randomized into four groups (PBS control, mIFN-β treatment, sorafenib treatment and sorafenib plus mIFN-β treatment). Sorafenib (30 mg/kg) was administered orally and mIFN-β (1 × 10^4^ IU/site) was administered via daily peritumoral injection for two weeks.

The tumors that developed in the mouse Hep3B cell-xenograft model were used for histological analysis including mitosis, apoptosis, and necrosis. To detect G2 to M phase cells, sections of the tumors were immunostained with a mouse anti-human topoisomerase IIα antibody (MBL Co., Ltd, Japan) and then with an anti-mouse immunoglobulin antibody. Immunoreacted cells were visualized with 3, 3′-diaminobenzidine (DAB). Topoisomerase IIα-positive cells were counted in 10 areas selected randomly in each specimen of tumors and were used for the evaluation of mitotic activity. To detect apoptotic cells, sections of the tumors were immunostained with a rabbit anti-single stranded DNA antibody (IBL Co., Ltd, Japan) and then with an anti-rabbit immunoglobulin antibody. Immunoreacted cells were visualized with HistoGreen. Single stranded DNA-positive cells were counted in 10 areas selected randomly in each specimen of tumors and were regarded as apoptotic cells. In addition, the sections were immunostained with a rat anti-mouse CD34 antibody (Santa Cruz Biotechnology, Inc. TX) or a rat anti-mouse CD31 antibody (BD Biosciences, CA) and then with an anti-rat immunoglobulin antibody. Immunoreacted cells were visualized with DAB. CD34- and CD31-positive cells were counted in 10 areas selected randomly in each specimen of tumors and were regarded as endothelial cells. As for CD31, immunoreacted cells were also visualized with fluorescence using Alexa Fluor546, and CD31-positive cells were counted in more than 8 areas selected randomly in each specimen of tumors. To evaluate necrosis, we used the Image J software^32^ and necrotic areas were measured in 10 sections randomly selected in each specimen of tumors.

All experimental procedures were approved by the Animal Care Committee of Hyogo College of Medicine and performed according to the guidelines issued by the National Academy of Science of Japan (“Guide for the Care and Use of Laboratory Animals”).

### DNA Chip and pathway analysis

In order to compare the add-on effects of mIFN-β with sorafenib, we used the tumors that developed in the mice treated with either sorafenib monotherapy or sorafenib plus mIFN-β combination therapy. Total RNA was isolated using the RNAqueous^Ⓡ^ kit (Ambion, Austin, TX, USA). cDNA fragments generated out of aliquots of total RNA (50 ng) were subjected to the DNA chip analysis according to previously described methods^33^, and gene expression profiling of the tumors was performed using a GeneChip Human Gene 1.0 ST Array (Affymetrix, Santa Clara, CA). Up- and downregulated pathways were determined using the BRB-Array Tools Version: 4.1.0. Functional class scoring was performed according to the Efron-Tibshirani’s GSA test^34^, and the pathways with p-values less than 0.05 were determined to be significantly up- or downregulated.

### Statistical analysis

The numerical data for the comparisons among three or more groups were analyzed using a non-repeated measurements ANOVA, and statistical significance was further examined with the Bonferroni correction. A P value of <0.05 was considered to be significant.

## Electronic supplementary material

Enomoto Supplementary information
