# Supplementary material for: The in vivo antitumor effects of type I-interferon against hepatocellular carcinoma: the suppression of tumor cell growth and angiogenesis
Source: Sci Rep. 2017 Sep 22;7:12189. doi: 10.1038/s41598-017-12414-3 (PMC5610170; doi:10.1038/s41598-017-12414-3)
Supplement: Supplementary file 1 — Enomoto Supplementary information [file 41598_2017_12414_MOESM1_ESM.pdf]

Supplementary information

Title: The *in vivo* antitumor effects of type I-interferon against hepatocellular carcinoma: the suppression of tumor cell growth and angiogenesis

Authors: Hirayuki Enomoto, Lihua Tao, Ryoji Eguchi, Ayuko Sato, Masao Honda, Shuichi Kaneko, Yoshinori Iwata, Hiroki Nishikawa, Hiroyasu Imanishi, Hiroko Iijima, Tohru Tsujimura, Shuhei Nishiguchi

Supplementary Figure S1: Effects of hIFN-β on the proliferation of human hepatocellular carcinoma HepG2 cells

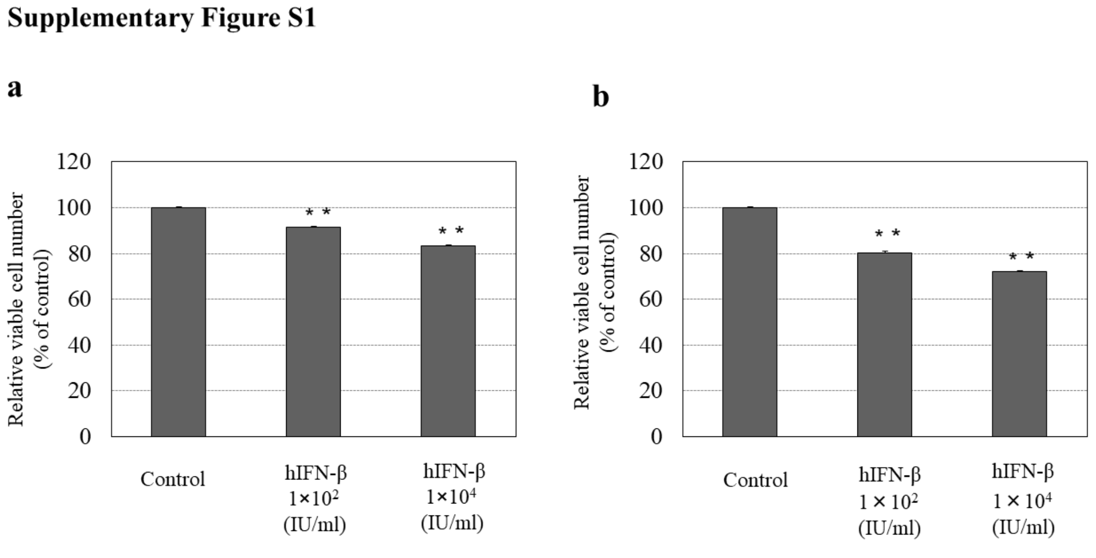

Human HCC HepG2 cells were cultured in DMEM containing 10% FBS in 5% CO_2 at

37°C and were treated with hIFN-β for 48 hours (a) or 72 hours (b). The proliferation of HepG2 cells was significantly inhibited with the hIFN-β treatment. **P<0.01 versus control (PBS treatment).

Supplementary Figure S2: *In vivo* antitumor effects of sorafenib plus mIFN-β treatment in the mouse xenograft model (HepG2 tumors).

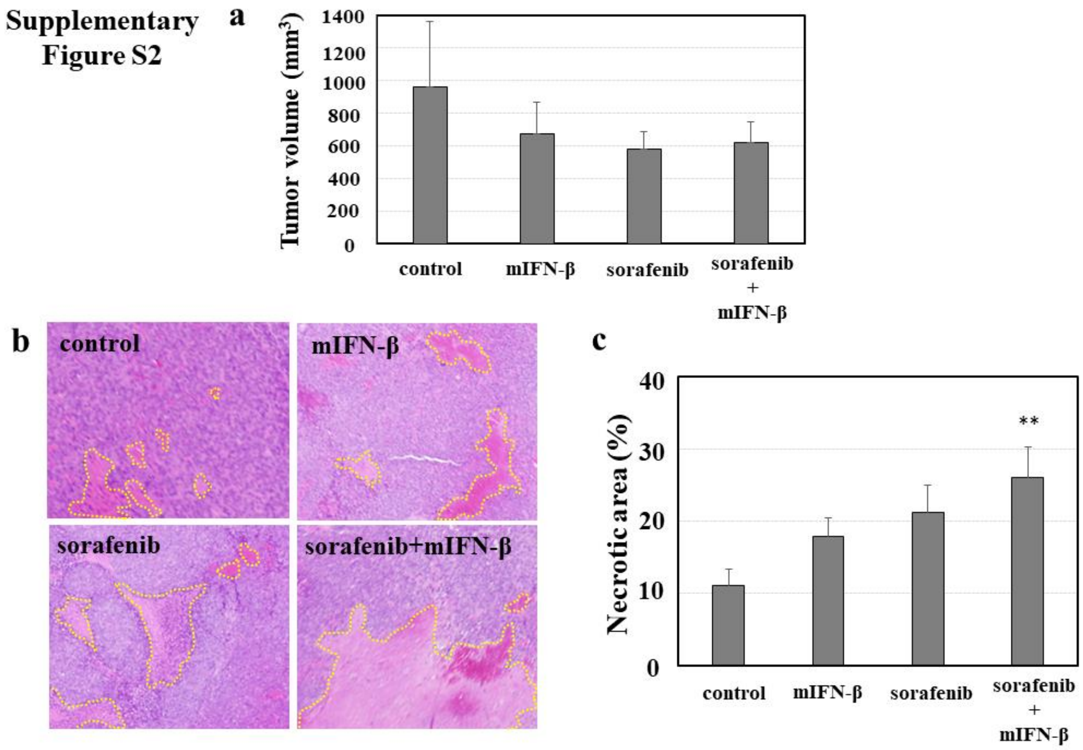

Five weeks after inoculation of HepG2 cells, tumor-bearing mice were randomized into four groups (PBS control, mIFN-β treatment, sorafenib treatment and sorafenib plus mIFN-β treatment) and were treated according to the procedures of **Fig. 8**. (a) Formed

tumor volume in the mouse HepG2 cell-xenograft model was not significantly different among the four groups. (b) Sections of HepG2 tumors were stained with hematoxylin-eosin, and the necrotic areas are surrounded by the dot lines. (c) Treatment with sorafenib plus mIFN-β resulted in increased the necrotic areas compared with the tumors that developed in the control (PBS treatment) mice (**P<0.01).

Supplementary Figure S3: *In vivo* anti-angiogenic effects of sorafenib plus mIFN-β treatment in the mouse xenograft model (HepG2 tumors)

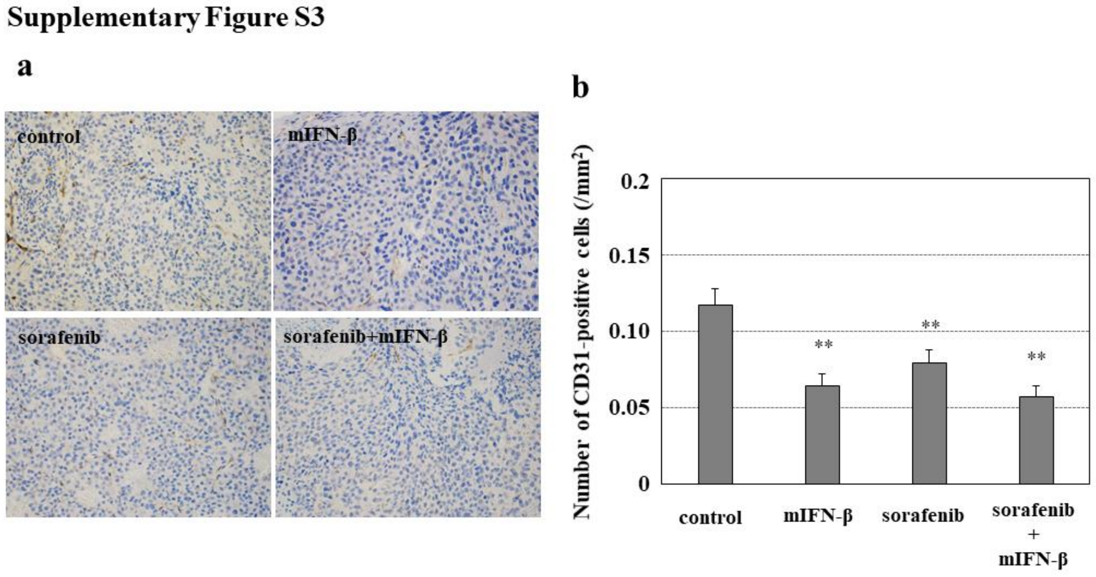

Sections of HepG2 tumors used in **Supplementary Fig. S2** were immunostained with an anti-mouse CD31 antibody according to the similar procedures to **Fig. 9**. (a) Sections of HepG2 tumors were immunostained with an anti-mouse CD31 antibody and positive

signals were visualized with DAB. Representative photographs are shown. (b) Sorafenib treatment, mIFN-β treatment and sorafenib plus mIFN-β treatment decreased the number of CD31-positive cells compared with the tumors that developed in the control (PBS treatment) mice (**P<0.01).

Supplementary Figure S4: Effects of hIFN-α and hIFN-β on the induction of apoptosis in HUVECs.

Supplementary Figure S4

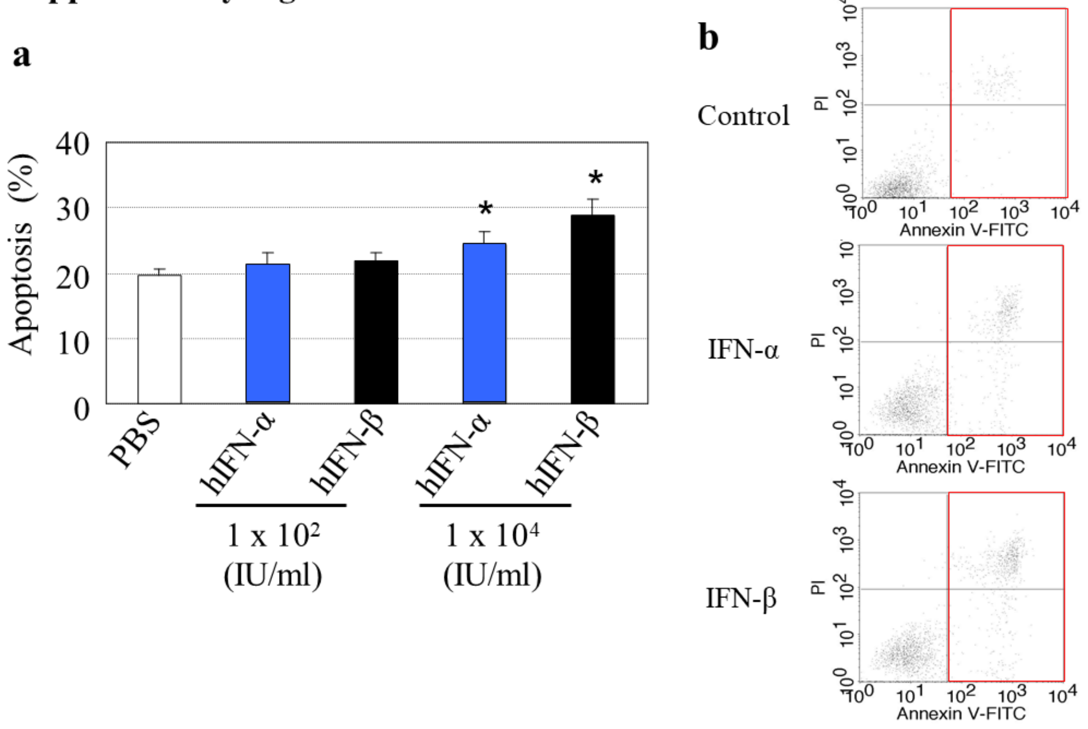

(a) HUVECs were cultured between two collagen gel layers in the presence of hIFN-α or hIFN-β, and stained with DAPI. Apoptotic cells exhibiting chromatin condensation

and/or nuclear fragmentation were counted, and the proportion of apoptotic cells to total cells is shown. Both hIFN-α and hIFN-β induced the apoptosis of HUVECs; however, the effects of hIFN-β treatment seems to be higher than those of hIFN-α treatment. *$P<0.05$ versus control (PBS treatment). (b) HUVECs were cultured in the medium for 24 hours in the presence of 1 x 10^4 IU/ml hIFN-α or hIFN-β. The cells were stained with annexin V/PI and analyzed using flow cytometry (FITC Annexin V Apoptosis Detection Kit II, BD Biosciences). Cells considered viable are annexin V^- and PI^-, cells in early apoptosis are annexin V^+ and PI^-, and cells in late apoptosis are annexin V^+ and PI^+. Apoptotic cells were detected in the areas surrounded with the red squares. Higher rates of apoptotic cells were observed in the cells treated with hIFN-β than in those treated with hIFN-α.

Supplementary Table 1.

Topo IIα-positive cells (count/mm²)				ssDNA-positive (count/mm²)			
**control**	**sorafenib**	**sorafenib+mIFN-β**	**P-value**	**control**	**sorafenib**	**sorafenib+mIFN-β**	**P-value**
397.3±13.0	381.3±12.9	384.7±14.6	NS	20.5±1.4	21.2±2.0	21.0±3.4	NS

Sections of Hep3B tumors were immunostained with an anti-human Topo IIα antibody and an anti-ssDNA antibody, and the number of proliferating (Topo IIα-positive) Hep3B cells and apoptotic (ssDNA-positive) Hep3B cells were counted.
Topo IIα, topoisomerase IIα; ssDNA, single stranded DNA
